# Small bowel stenosis after blunt abdominal trauma: a case report

**DOI:** 10.1186/s40792-020-00874-1

**Published:** 2020-05-26

**Authors:** Kazushi Hara, Manabu Yamamoto, Teruhisa Sakamoto, Ken Sugezawa, Chihiro Uejima, Akimitsu Tanio, Yoichiro Tada, Takehiko Hanaki, Kozo Miyatani, Joji Watanabe, Kyoichi Kihara, Naruo Tokuyasu, Shuichi Takano, Soichiro Honjo, Yoshiyuki Fujiwara

**Affiliations:** Division of Surgical Oncology, Department of Surgery, School of Medicine, Tottori University Faculty of Medicine, 36-1 Nishi-cho, Yonago, 683-8503 Japan

**Keywords:** Blunt abdominal trauma, Ileus, Laparoscopic surgery, Small bowel stenosis

## Abstract

**Background:**

Small bowel stenosis after blunt abdominal trauma is relatively rare, and progression from trauma to bowel stenosis might sometimes be delayed. Herein, we report the case of a patient who was diagnosed with small bowel stenosis relatively early and received laparoscopic surgery.

**Case presentation:**

An 18-year-old Japanese male was in a traffic accident and was urgently transported to our hospital. On arrival, he was admitted with right kidney and right adrenal injury and abdominal aortic aneurysm. On hospital day 13, he vomited during conservative treatment without surgery, and computed tomography revealed small bowel stenosis and dilatation of the oral-side small bowel. No improvement with the ileus tube occurred, and he received laparoscopic surgery on hospital day 21. Briefly, the abdominal cavity was observed with a laparoscope. The mesentery was congested, scarring around the stenotic small bowel regions was present, and three stenotic regions were observed 40–50 cm from the Treitz ligament. The patient received partial resection and anastomosis of the small bowel. The postoperative course was stable, and he was discharged on postoperative day eight.

**Conclusions:**

Most cases of bowel stenosis after abdominal trauma are irreversible and usually require surgical treatment. Therefore, small bowel stenosis should be considered in patients with abdominal symptoms after blunt abdominal trauma.

## Background

Gastrointestinal damage due to blunt abdominal trauma often presents with symptoms immediately after injury due to organ rupture or bowel perforation and requires emergent surgery. However, patients rarely develop small bowel stenosis or obstruction several days to months after the initial trauma. Herein, we report the case of a patient with small bowel stenosis on day 13 after blunt abdominal trauma who was successfully treated with partial small bowel resection by laparoscopic assistance.

## Case presentation

**Fig. 1:**
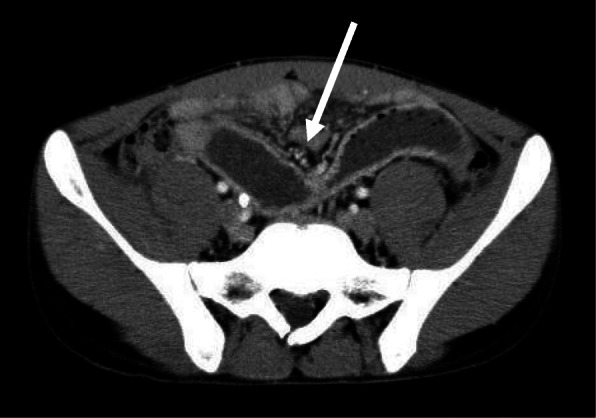
CT showing stenosis of the small bowel and dilatation of the oral small bowel

**Fig. 2:**
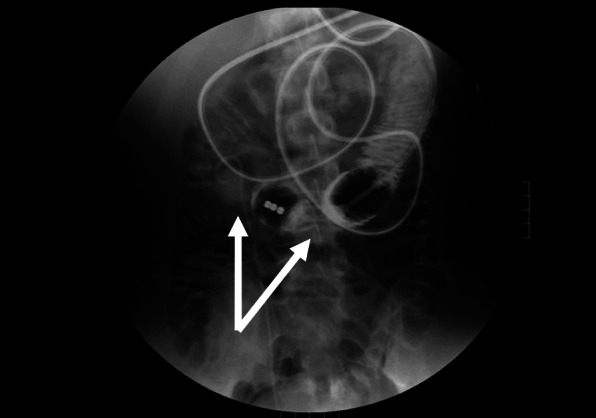
Gastrointestinal contrast study showing at least two stenotic areas in the small bowel (white arrows)

An 18-year-old Japanese male patient was urgently transported to our hospital following a traffic accident. The patient was sitting in the passenger seat with the seat belt fastened and had a car-to-car accident. The patient was diagnosed with right kidney injury, right adrenal injury, and abdominal aortic aneurysm and was admitted to the hospital. Intra-abdominal hemorrhage due to renal injury was controlled by endovascular treatment. The abdominal aortic aneurysm was followed without any intervention. Other organs, including the small bowel, did not show any damage on the first computed tomography (CT) examination. The clinical course of the patient was good after these treatments, and food intake was initiated on the tenth hospital day. However, on hospital day 13, the patient vomited bile-like fluid twice. He experienced no abdominal pain, and hematological tests did not reveal any abnormal findings. CT showed stenosis and oral-side dilatation of the small bowel (Fig. 1), and he was diagnosed with small bowel obstruction. Although he was conservatively treated with fasting and infusion initially, the CT findings on hospital day 19 did not improve the bowel obstruction; therefore, an ileus tube was placed. The next day, gastrointestinal contrast examination using the ileus tube revealed at least two stenotic regions in the small bowel (Fig. 2). Based on these findings, the patient was diagnosed with small bowel stenosis due to blunt abdominal trauma and received laparoscopic surgery on hospital day 21.

**Fig. 3:**
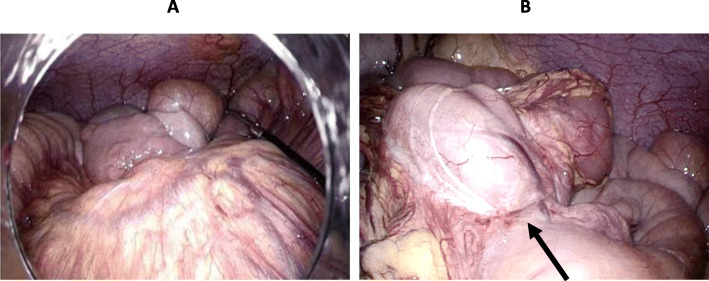
Intraoperative findings. **a** Small bowel mesentery is congested. **b** Note stenosis in the small bowel (arrow)

First, a 12-mm port was placed using the open method in the umbilical site and the abdominal cavity was observed with a laparoscope. A localized area of the mesentery was congested and edematous, and scarring around the stenotic section of the small bowel was present (Fig. 3a, b). A small incision was made in the umbilicus, and the stenotic sections of the small bowel were brought outside through the abdominal wall. Close observation revealed three stenotic regions which were 40–50 cm from the Treitz ligament, and partial resection and anastomosis of the small bowel were performed. The operation time was 2 h and 20 min, and the blood loss was 5 mL.

**Fig. 4:**
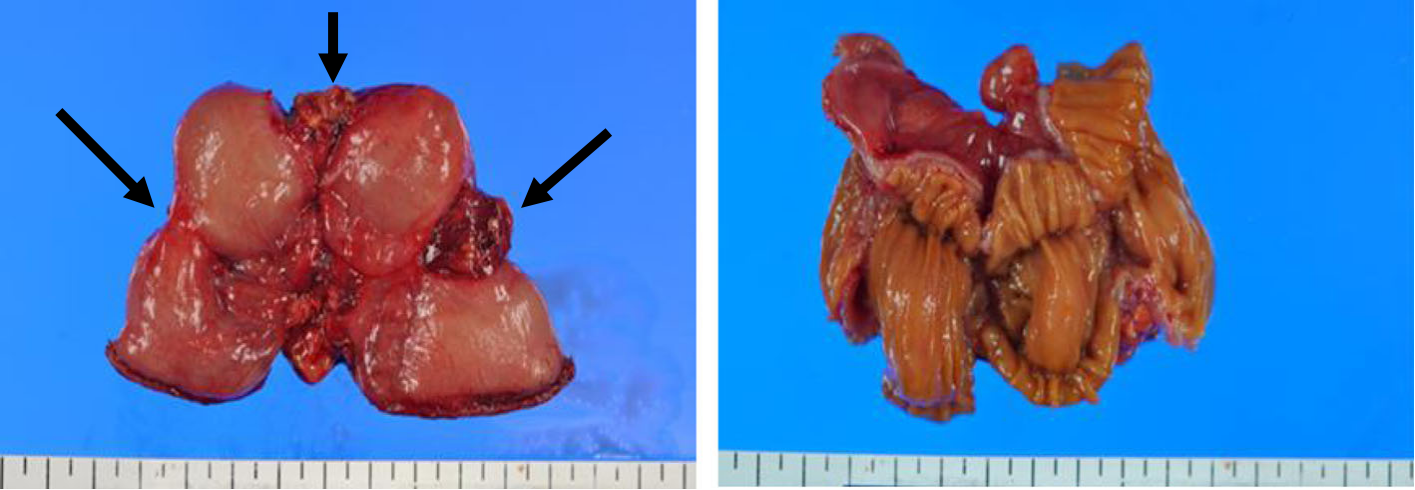
Resected specimen showing three areas of stenosis

**Fig. 5:**
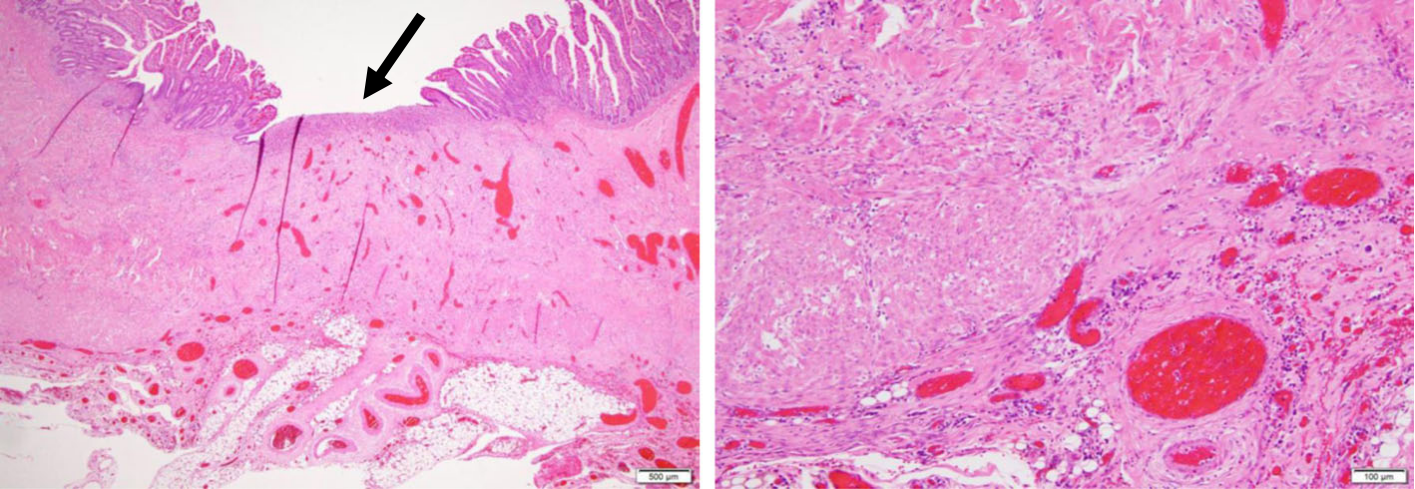
Histological findings. **a** Mucosal erosion and ulceration are observed (arrow), and the muscle layer is destroyed and ruptured just below. **b** Capillary growth and dilatation and fibrosis can be observed

The excised specimen showed three stenotic regions (Fig. 4). The pathological examination revealed erosion of the mucosa and ulceration; the muscle layer was also destroyed and partially ruptured (Fig. 5a). The strong expansion and hyperplasia of the capillaries, which were recognized as findings of fibrosis, were also observed (Fig. 5b).

The postoperative course was stable, and the patient was discharged on postoperative day eight.

## Discussion

Patients with gastrointestinal damage due to blunt abdominal trauma often present with acute symptoms due to bowel rupture and/or bleeding, usually requiring emergent surgery. However, in rare cases small bowel stenosis or obstruction occurs several days or months after the trauma [1–3]. The underlying causes of trauma-induced intestinal stenosis include altered circulation due to mesenteric damage, direct damage to the intestinal wall and hematoma formation, and inflammatory adhesions [4]. Mesenteric damage causes injury to the mesenteric artery and leads to necrotic changes and ulceration of the intestines due to altered blood flow. During healing, stenosis occurs due to scar contraction. The blood flow dysfunction usually includes the entire intestinal wall circumference, leading to annular stenosis [5]. In the present case, edematous changes were observed in the mesentery, and histological examination showed fibrosis with ulceration and infiltration of the inflammatory cells. Small bowel stenosis was therefore considered to arise from the altered circulation due to mesenteric damage and subsequent scar formation. Given that intestinal stenosis is usually irreversible due to scarring and fibrosis, conservative treatments such as decompression are not applicable, and surgery is required in most cases.

In our case, we performed laparoscopic surgery. If the surgery needs to be performed within a short period after the injury, open surgery may be chosen because of the possibility of damage to other organs and the need for a detailed intra-abdominal search. However, in our case, it had been several days since the injury, and a diagnosis of only small bowel stenosis was made, with no other abnormalities; furthermore, decompression of the small intestine was successful using an ileus tube. Therefore, we selected laparoscopic surgery. The 15-year-old patient underwent partial resection of the small intestine with a 3-cm incision; it was less invasive, and laparoscopic surgery was also useful in terms of integrity.

Bowel stenosis due to abdominal trauma is often located near the body midline such as the upper small bowel and terminal ileum, where the mesentery is relatively fixed [6–8]. Except for the small intestine, stenosis is commonly observed in the duodenojejunal flexion, transverse colon, and sigmoid colon, which are close to the spine, suggesting that gastrointestinal damage might be due to the compression of the affected area between the abdominal wall and spine [9].

**Fig. 6:**
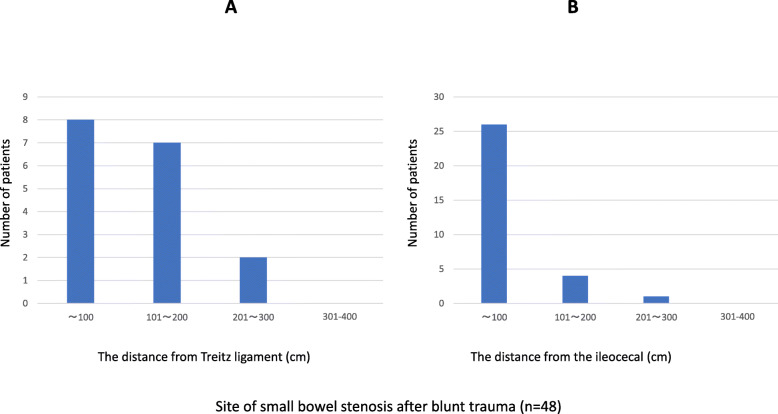
Site of the small bowel stenosis after blunt trauma (*n* = 54). **a** Distance from Treitz ligament to the small bowel stenosis. **b** Distance from the ileocecal valve to the small bowel stenosis

**Fig. 7:**
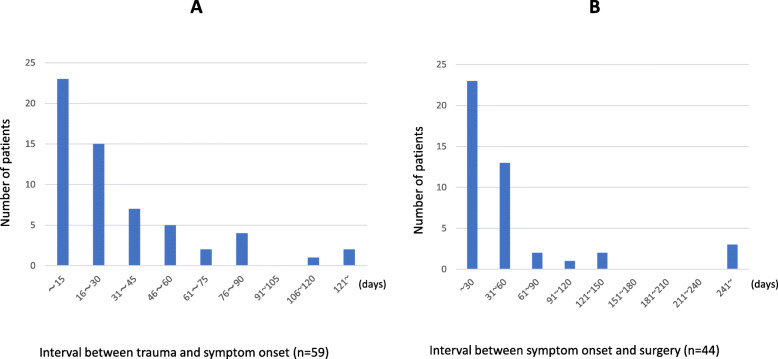
**a** Interval between trauma and symptom onset. **b** Interval between symptom onset and surgery

We searched the literature from 1980 to 2018 using the keywords “abdominal trauma,” “late onset,” and “small bowel stenosis” using the PubMed and the Japan Medical Abstracts Society electronic databases, and the 62 collected cases were reviewed, 47 males and 15 females with an average age of 40.5 (5–80) years. The cause was traffic accidents in 41 of these 62 cases. In 87% of the cases, the location of delayed small bowel stenosis was within 100 cm from the ileocecal region and within 200 cm from the Treitz ligament (Fig. 6). The location of the stenosis was also 40–50 cm from the Treitz ligament in the present case, consistent with the previous reports. The interval from trauma to symptom onset was within 15 and 30 days in 23 (39%) and 38 (64%) of the cases in the literature, respectively, whereas stenosis occurred after 60 days in 9 (15%) cases. While 23 (52%) patients received surgery within 30 days from the symptom onset, 21 (48%) patients did not receive surgery even after the first 30 days (Fig. 7).

Several reasons underlie delayed diagnosis and surgery in these patients. First, temporal conservative treatment might improve symptoms in patients with incomplete stenosis. Additionally, since symptom development takes time, the patient might be handled without the knowledge of prior blunt abdominal trauma if they are treated for stenosis-related symptoms in a hospital other than the emergency hospital handling the initial trauma. Therefore, obtaining detailed medical history on abdominal trauma is important to ensure early diagnosis.

## Conclusions

Post-traumatic intestinal stenosis should be considered in patients with a history of blunt abdominal trauma presenting with abdominal symptoms.

## Data Availability

All data regarding this paper are available on request.

## Abbreviations

CT: Computed tomography
